# Cytotoxic activity of the genus *Ferula *(Apiaceae) and its bioactive constituents

**Published:** 2018

**Authors:** Mehrdad Iranshahi, Ramin Rezaee, Mona Najaf Najafi, Ali Haghbin, Jamal Kasaian

**Affiliations:** 1 *Biotechnology Research Center, School of Pharmacy, Mashhad University of Medical Sciences, Mashhad, Iran*; 2 *Clinical Research Unit, Faculty of Medicine, Mashhad University of Medical Sciences, Mashhad, Iran*; 3 *Natural Products and Medicinal Plants Research Center, North Khorasan University of Medical Sciences, Bojnurd, Iran*

**Keywords:** Apiaceae, Ferula, Biological activity, Cytotoxicity, Umbelliprenin, Sesquiterpene coumarin, Farnesiferol C

## Abstract

**Objective:**

The genus *Ferula* L. includes perennial flowering plants belonging to the Apiaceae family. This genus is a rich source of biologically active phytochemicals such as sulfur-containing derivatives, coumarins, sesquiterpenes, sesquiterpene lactones, sesquiterpene coumarins, glucuronic acid, galactose, arabinose, rhamnose, and daucane esters. Over the last decade, considerable attention has been paid to biological activities of these compounds; it is assumed that the most prominent biological features of the genus *Ferula *are their cytotoxic effects. This article discusses cytotoxic activity of the genus *Ferula *and their important compounds.

**Materials and Methods:**

In this mini-review article, papers published from 1990 to April 2016 were included and the following information was discussed; cytotoxic activity of the genus *Ferula *and their important compounds, the type of cell line used *in vitro*, concentrations of the extracts/active compound that were used, and the underlying mechanisms of action through which *Ferula*-related chemicals induced cytotoxicity. In addition, we explained different mechanisms of action through which the active constituents isolated from *Ferula*, could decrease cellular growth.

**Conclusion:**

It is highly recommended that potent and effective compounds that were isolated from *Ferula *plants and found to be appropriate as adjuvant therapy for certain diseases, should be identified. Also, the versatile biological activities of sesquiterpene coumarins suggest them as promising agents with a broad range of biological applications to be used in the future.

## Introduction

**Figure1:**
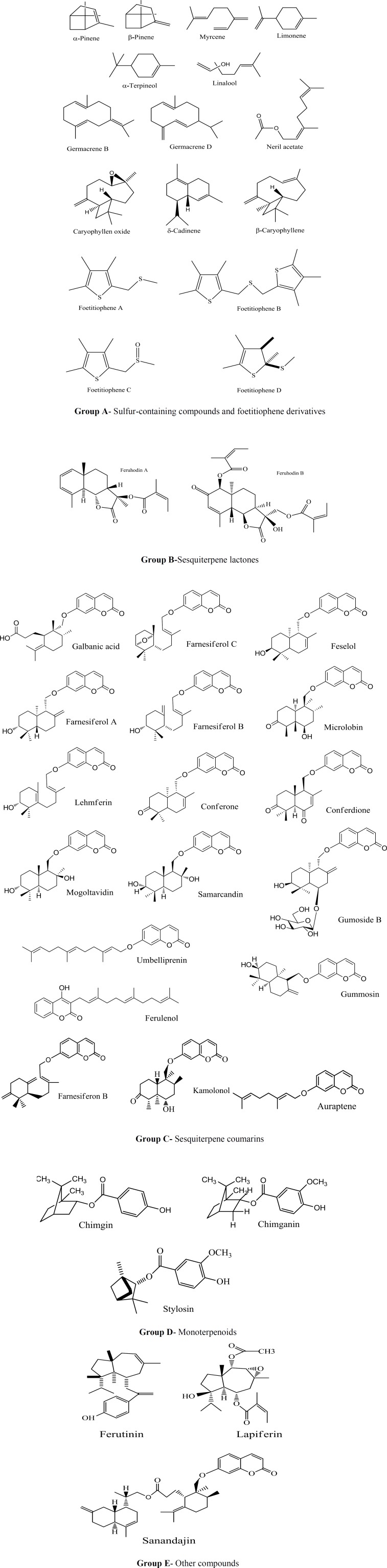
Chemical structure of some constituents of *Ferula *categorized in groups A-E

The genus *Ferula *includes perennial flowering plants belonging to the family Apiaceae (Umbelliferae). This genus consists of about170 species which are distributed worldwide. Out of 30 species of *Ferula *that could be found in Iran, 16 plants are endemic. Different species of the genus *Ferula *are broadly distributed in arid areas from the eastern Mediterranean regions to central Asia (Gholami and Shamsara, 2016 ▶; Karimi et al., 2010 ▶; Nazari and Iranshahi, 2011 ▶); however, some *Ferula *species are found in arid regions of temperate Eurasia, in the Canary Islands and in North Africa (e.g. Tunisia) (Znati et al., 2014 ▶). Different species of the genus *Ferula *are regarded as rich sources of biologically active phytochemicals such as sulfur-containing derivatives, coumarins, coumarin esters, sesquiterpenes, sesquiterpene lactones, sesquiterpene coumarins, glucuronic acid, galactose, arabinose, rhamnose, and daucane esters (Figure. 1) (Asghari et al., 2016 ▶; Maggi et al., 2016 ▶; Nazari and Iranshahi, 2011 ▶; Razavi et al., 2016 ▶).

Some species of the genus *Ferula *have therapeutic properties such as contraceptive, antipyretic, smooth-muscles relaxant and aphrodisiac activities (Nazari and Iranshahi, 2011 ▶; Yaqoob et al., 2016 ▶). Also, several *Ferula* species are well-known because of their applications in the treatment of various diseases. For example, *F. persica* root extract possesses antispasmodic, carminative, laxative and expectorant properties and has been used for the treatment of diabetes and high blood pressure (Razavi and Janani, 2015 ▶). *F. assa-foetida* exhibits anti-carcinogenic properties and has protective activities against free radical-mediated diseases (Gamal-Eldeen and Hegazy, 2010 ▶). Iranshahi et al. reported that *F. assa-foetida *has anti-leishmanial activity against promastigotes (Iranshahi et al., 2007 ▶). Moreover, *Ferula* species have been used in traditional medicine for the treatment of skin infections, hysteria and stomach disorders. Also, a number of *Ferula* species has been utilized as febrifuge and carminative agents and for relaxation of tracheal smooth muscles (Gamal-Eldeen and Hegazy, 2010 ▶). *F. assa-foetida* and *F. gummosa *are two famous species of *Ferula *in Iranian folk medicine*. *Additionally, some* Ferula* species are well-known as important sources of aromatic resins and are employed in cosmetic industries (Kanani et al., 2011 ▶).

Phytochemicals obtained from the species of *Ferula* are used in traditional medicine for the treatment of various diseases such as digestive disorders, rheumatism, headache, neurological disorders, arthritis, dizziness and dysentery. Galbanum, the aromatic gum resin obtained from *F. gummosa*, has been traditionally used as a tonic, anticonvulsant, and emmenagogue agent (Iranshahi et al., 2010 ▶). Moreover, as asafoetida as the dried latex (gum oleoresin) exudates from the rhizome or tap root of *F. assa-foetida*, has been traditionally used for the treatment of various diseases including asthma and gastrointestinal disorders as well as removal of intestinal parasites. Asafoetida has also been known to possess antifungal, anti-diabetic, anti-inflammatory, anti-mutagenic and antiviral activities (Iranshahy and Iranshahi, 2011 ▶; Mahendra and Bisht, 2012 ▶).

A number of sesquiterpenes obtained from the species of* Ferula* roots, revealed antibacterial, antifungal, cytotoxic, antioxidant, and hormonal activities as well as P-glycoprotein inhibitory and immunomodulatory effects (Miski, 2013 ▶). Sanandajin and ethyl galbanate, the two sesquiterpene coumarins isolated from *F. pseudalliacea* root extract have shown potent antibacterial activities and are being used in pharmaceutical and food industries (Dastan et al., 2016 ▶).

In this review, we focused on cytotoxic activity of *Ferula *plants reported from 1990 to April 2016.

**Cytotoxicity**

Ferulenol, a prenylated 4-hydroxycoumarin isolated from *F. communis,* exerted dose-dependent cytotoxicity against various human tumor cell lines. It stimulated tubulin polymerization *in vitro*, inhibited the binding of radio-labeled colchicine to tubulin, re-arranged cellular microtubule network into short fibres and altered nuclear morphology (Bocca et al., 2002 ▶). In another study, the cytotoxicity of ferulenol on human breast cancer (MCF-7), colon cancer (Caco-2), ovarian cancer (SKOV-3) and leukemic (HL-60)cells was evaluated; based on the results, ferulenol showed significant cytotoxic effects at concentrations of 10 nM, 100 nM and 1µM, against these cancer cell lines (Nazari and Iranshahi, 2011 ▶). Conferone is another sesquiterpene comarin isolated from *Ferula *root extract. Barthomeuf et al. (2006) ▶ showed that 10μM of conferone enhances the cytotoxicity of vinblastine in MDR1-transfected Madin-Darby canine kidney (MDCK-MDR1) cells (Barthomeuf et al., 2006 ▶). Additionally, conferone enhanced the cytotoxicity of cisplatin and vincristine in 5637 cells (Neshati et al., 2012 ▶; Neshati et al., 2009 ▶). In another study, conferone exhibited moderate cytotoxicity against CH1 (human ovarian carcinoma) and A549 (human nonsmall cell lung cancer) cells (Valiahdi et al., 2013 ▶). Also, umbelliprenin, a prenylated coumarin synthesized by various *Ferula* species, showed cytotoxic activity by inhibition of the growth of human M4Beu metastatic pigmented malignant melanoma cells through induction of cell cycle arrest in G1 and caspase-dependent apoptosis (Lourenco et al., 2012 ▶). Khaghanzadeh et al. (2012) ▶ studied umbelliprenin cytotoxicity in two different types of lung cancer cell lines (i.e. QU-DB and A549). Their results revealed that IC_50_ values for QU-DB and A549 were 47±5.3 and 52±1.97 μM, respectively (Khaghanzadeh et al., 2012 ▶). Also, an investigation on umbelliprenin nanoliposomes revealed that liposomal umbelliprenin possesses time and concentration-dependent cytotoxicity on melanoma cell line (Ramezani et al., 2014 ▶). Additionally, umbelliprenin showed antigenotoxic properties in human peripheral lymphocytes, probably due to its prenyl moiety (Soltani et al., 2009 ▶). In another investigation, auraptene, a prenylated coumarin isolated from *Ferula, *exerted cytotoxic effects against MCF-7cell line (IC_50_=59.7 µM) (Mousavi et al., 2015 ▶).

Furthermore, stylosin and tschimgine (monoterpenes isolated from *Ferula ovina*) showed cytotoxic activities against human melanoma cell line SK-MEL-28 (Valiahdi et al., 2013 ▶). Also, Rassouli et al. (2011) ▶ reported the cytotoxic and apoptosis-inducing effects of stylosin (Rassouli et al., 2011 ▶).

Feselol and mogoltacin are two biologically active sesquiterpene coumarins isolated from root extracts of* Ferula* species that showed cytotoxic properties. For example, a combination of 40 mg/mL vincristine and 16 mg/mL mogoltacin increased the cytotoxicity of vincristine by 32.8%, in human transitional cell carcinoma (TCC) cells (BehnamRassouli et al., 2009). Similar results were found for feselol, a sesquiterpene coumarin isolated from the fruits of *F. badrakema *(Mollazadeh et al., 2010 ▶). Also, a combination of feselol and mogoltacin enhanced the cytotoxicity of cisplatin in 5637 cells (human bladder carcinoma cell line) (Mollazadeh et al.,2011 ▶; Rassouliet al., 2011 ▶). Hanafi-Bojd et al. (2011) ▶ showed that farnesiferol A and galbanic acid, two sesquiterpene coumarins isolated from *Ferula* species, increase verapamil cytotoxicity (Hanafi-Bojd et al., 2011 ▶). In another study, sanandajin, farnesiferol B, and kamolonol acetate displayed cytotoxic activities against HeLa cells with IC_50 _values of 2.2, 6.7, and 4.9 μM, respectively (Dastan et al., 2014 ▶). Kasaian et al. (2015) ▶ revealed that sesquiterpene coumarins isolated from *Ferula* species exert different cytotoxic activities. Also, they reported that farnesiferol B, farnesiferol C and lehmferin reverse doxorubicin-resistance properties of MCF-7/Adr cells (Kasaian et al., 2015 ▶).

Methyl caffeate, a compound isolated from *F. lutea *showed cytotoxic effects, with IC_50_ values of 22.5±2.4, 17.8±1.1 and 25±1.1 μmol/L against HCT-116 (human colon carcinoma cell line), IGROV-1 and OVCAR-3 (human ovarian cancer cell line), respectively (Znati et al., 2014 ▶). Also, kamolonol, 4′-hydroxy kamolonol acetate and farnesiferon B, the three sesquiterpene coumarins isolated from the roots of *F. pseudalliacea*, displayed cytotoxic activity against HeLa cells, with IC_50_values of 3.8, 4.5, and 7.7 μM, respectively (Dastan et al., 2014 ▶). However, Ghannadi et al. (2014 ▶) reported that kellerin, an active compound of *F. assa-foetida*, had no cytotoxic effect against Vero cells up to the concentration of 10 µg/mL (Ghannadi et al., 2014 ▶). Galbanic acid, the other sesquiterpene coumarin isolated from *F. szowitsiana*, inhibited A549 growth with an IC_50_ value of 62 μM following 48hr treatment (Eskandani et al., 2015 ▶).

Chitsazian-Yazdi et al. (2015) ▶ investigated 4 new foetithiophene compounds namely, foetithiophene C, foetithiophene D, foetithiophene E and foetithiophene F isolated from *F. foetida*. They revealed that these compounds have no significant cytotoxic activities (IC_50_ values>100 mM) against MCF-7 and K562 cancer cells (Chitsazian-Yazdi et al., 2015 ▶). Ferutinin is a natural product isolated from *F. ovina* possesses apoptosis-inducing effects. Also, ferutinin analogues synthesized by esterification of jaeschkenadiol using different acids, have exhibited potent inhibitory activity against MCF-7 with an IC_50_ value of 1 μm (Matin et al., 2014 ▶; Safi et al., 2015 ▶).

A number of sesquiterpene lactones isolated from *F. oopoda* showed significant cytotoxicity. For example, dehydrooopodin revealed significant cytotoxicity with IC_50_ values of 5 and 15 µM against K562 and MCF7cancer cell lines, respectively (Kasaian et al., 2014 ▶).

Moreover, the cytotoxicity of dehydrooopodin and oopodin, two sesquiterpene lactones isolated from *F. varia *were tested against KB (human epidermoid carcinoma of the nasopharynx), K562 (leukemia), MCF7, and COLO 205 (coloncarcinoma) cell lines, as well as the multidrug-resistant human cancer cell lines KB-C2 (colchicine-resistant KB) and K562/ADR (Adriamycin-resistant K562). These compounds showed moderate cytotoxicity with IC_50_ values ranging from 24.7 to 56.9 μg/mL (Suzuki et al., 2007 ▶).

Cytotoxicity of some sesquiterpene coumarins isolated from *F. sinkiangensis* was investigated by Li et al., 2015 ▶. They found that these sesquiterpene coumarins had selective cytotoxic activity against HeLa and AGS cancer cell lines, with IC_50_ values of 12.7-226.6 μM (Li et al., 2015 ▶).

In 2006, it was reported that compounds isolated from *F. assa-foetida* have potent and specific NF-κB-inhibiting properties, but their cytotoxicity were negligible (Appendino et al., 2006 ▶). 

Chimgin and chimganin, two monoterpenoid compounds isolated from *F. szowitsiana*, showed cytotoxic activities. Chimgin showed IC_50 _values of 45.2, 67.1 and 69.7 µM and chimganin showed IC_50_values of 28, 74 and 30.9 μM for MCF-7, HepG2 and MDBK cancer cell lines, respectively. These values were just slightly lower than those of tamoxifen which was used as positive control (Sahranavard et al., 2009 ▶).

In a number of investigations, *Ferula* root extracts and fractions have been studied. Eslami et al. (2015) ▶ showed that *F. gummosa* extract has specific cytotoxic effects mainly against MCF7 and oral cancer cell lines (Eslami et al., 2013 ▶; Gudarzi et al., 2015 ▶). Elouzi et al. (2008) ▶ proved that petroleum extract of *F. hermonis*at the concentration of 0.125 mg/ml, causes 50% cell death (Elouzi et al., 2008 ▶). 

The extract of *F. szowitsiana* root was shown to be active against three cancerous (MCF7, HepG2 and WEHI164) and one normal (MDBK) cell lines. In another study, the cytotoxicity of some of the Iranian medicinal *Ferula* species was examined and all the extracts and oleo-gum resins of *F. assa-foetida* showed dose-dependent cytotoxicity (Bagheri et al., 2010 ▶). 

Hajimehdipoor et al. (2012) ▶ investigated the cytotoxic effects of *F. persica* and *F. hezarlalezarica,* two endemic *Ferula* species of Iran, against MCF7, HepG2, HT29 and A549 (adenocarcinomic human alveolar basal epithelial cells), cancer cell lines. They revealed that hexane and chloroform fractions of these plants have cytotoxic effects at concentration up to 100 μg/ml. They also reported that the cytotoxicity of *F. persica* extracts was higher than that of *F. hezarlalezarica *extracts (IC_50_: 22.3-71.8 μg/ml for *F. persica* and 76.7-105.3 μg/ml for *F. hezarlalezarica*) (Hajimehdipoor et al., 2012 ▶).

 In an investigation, *F. assa-foetida* extract displayed neuroprotective effects in a glutamate-induced neurotoxicity model (Tayeboon et al. 2013 ▶). In another study, researchers reported the cytotoxic activities of the extracts and fractions of *F. szowitsiana*, *F. hirtella* and *F. oopoda *against MCF-7, HT-29, A549 and HepG2 cancer cell lines. Based on their data, n-hexane and chloroform fractions of *F. szowitsiana* and *F. hirtella* were cytotoxic, probably due to the presence of non-polar/semi-polar constituents (Hamzeloomoghadam et al. 2013 ▶). Furthermore, the cytotoxic properties of the *n*-butanol extract of *F. lutea* with an IC_50_=40 μg/ml against K562 (leukemia cell line) was reported (Znati et al., 2014 ▶).

The cytotoxicity of *F. assa-foetida* extract on HOS CRL,an osteosarcoma cell line was also investigated. The results of this investigation showed that the cytotoxic activity of *F. assa-foetida *extract is dependent on the type and concentration of the solvent. Moreover, the methanol extract possessed more marked cytotoxic effects than the ethanol extract (Shafri et al., 2015 ▶). In another study, results of MTT assay of *F. assa-foetida* extract against an osteosarcoma cell line (HOSCRL-1543) showed that this activity is dependent on the type of solvent (methanolic>ethanolic) and its concentration (higher methanolic content>lower methanolic content) (MohdShafri et al., 2015 ▶).

Gudarzi et al. (2015) ▶ showed anti-proliferative activity of ethanolic extract of *F. gummosa *seed, which was probably related to the presence of bioactive compounds like coumarins and terpenoids (Gudarzi et al., 2015 ▶). Additionally, cytotoxicity of hydroalcoholic extract of *F. gummosa* root was investigated on GP-293 cell line and primary cultured human stromal-vascular cells. The viability of human stromal-vascular cells following treatment with *F. gummosa* extract 400 mg/mL (60±6.5% of the control, p<0.01) and 800 mg/mL (14±1% of control, p<0.001) were significantly decreased. Also, the *F. gummosa* root extract reduced the viability of GP-293 cells at concentration of 750 mg/mL (8.8±0.35%, p<0.001) (Ghorbani et al., 2016 ▶).

**Some other cell-based assays**

Umbelliprenin and auraptene, two prenylated coumarins isolated from *F. szowitsiana *revealed cytotoxic properties. Umbelliprenin showed the highest inhibitory activity against M4Beu melanoma cell line (IC_50_=12.4±0.5 µM) compared to cisplatin (23.1±0.8 µM) (Paydar et al., 2013 ▶; Shakeri et al., 2014 ▶). Ziai et al. (2012) ▶ studied apoptosis-inducing activities of umbelliprenin in Jurkat T-CLL and Raji B-CLL cell lines. Their results showed that umbelliprenin induced apoptosis in leukemic cells in a dose- and time-dependent manner; also, CLL (Chronic lymphocytic leukemia) cells were more susceptible to umbelliprenin-induced cell death as compared to normal peripheral blood mononuclear cells (PBMCs) (Ziai et al., 2012 ▶). In another study, Barthomeuf et al. (2008 ▶) showed that umbelliprenin induces caspase-dependent apoptosis (IC_50_=12.3 µM) (Barthomeuf et al., 2008 ▶). Gholami et al. (2013) ▶ investigated the effect of umbelliprenin on pro-apoptotic caspases (caspase-8 and -9) and anti-apoptotic Bcl-2 family protein in Jurkat cell line. They revealed that umbelliprenin activates intrinsic and extrinsic pathways of apoptosis by activation of caspase-8 and caspase-9, respectively. They also found that umbelliprenin inhibits Bcl-2 protein. Furthermore, umbelliprenin induced apoptosis in Jurkat cells through a caspase-dependent pathway (Gholami et al., 2013 ▶).

Ferulenol, a prenylated coumarin from *F. communis* (Umbelliferae) exhibited tubulin-polymerizing activity. Under Ca^2+^-free conditions, ferulenol appeared to be equipotent as Taxol in promoting tubulin assembly (Altmann and Gertsch, 2007 ▶). Recently, it was shown that conferone 20 µM induces cell arrest and cell death through both apoptosis and necrosis in HT-29 cells (Cheraghi et al., 2016 ▶).

Galbanic acid, a sesquiterpene coumarin isolated from *Ferula* species showed cytotoxic activities. Galbanic acid inhibited the growth of prostate cancer cells via decreasing androgen receptor abundance (Kasaian et al., 2014 ▶). Also, galbanic acid induced apoptosis in H460 cells via caspase activation and Mcl-1 inhibition in H460 cells; therefore, it could be considered a potent cytotoxic agent against non-small cell lung carcinoma (Oh et al., 2015 ▶). Researchers also revealed that galbanic acid has anti-angiogenesis effects (Kim et al., 2011 ▶). 

Diversin, a natural prenylated coumarin isolated from *Ferula* roots, revealed cytotoxic activity as well as cell-cycle-inhibitory and apoptosis-inducing effects on bladder carcinoma cells (Haghighitalab et al., 2014 ▶).

Umbelliferone, a naturally occurring coumarin derivative isolated from *F. communis*, has been suggested as an effective cytotoxic compound against HepG2 cell line. Furthermore, umbelliferone exhibited apoptosis-inducing activity in HepG2 cells in a concentration-dependent manner (0-50 μM) (Yu et al., 2015 ▶).

Huang et al. (2013) ▶ investigated two new terpenoid benzoates namely, syreiteate A and syreiteate B, isolated from the roots of *F. dissecta. *Their results proved that syreiteate A and syreiteate B have potent growth inhibitory activity against cervical cancer HeLa cell line with IC_50_ values of 13.2 and 19.3 μM, respectively (Huang et al., 2013 ▶).

Ferutinin, a natural sesquiterpene of *Ferula*, showed apoptosis-inducing activities in cancerous cells by induction of sub-G1 peak as revealed by PI staining (Arghiani et al., 2014 ▶). Researchers also showed that ferutinin has apoptotic effects in human Jurkat T-cell line (Macho et al., 2004 ▶). 

Nano-based formulation of farnesiferol C, a sesquiterpene coumarin isolated from *Ferula*, significantly suppressed the proliferation of AGS gastric epithelial cells in a time- and dose-dependent manner (p<0.01). Farnesiferol C could be considered a potential chemotherapeutic agent; its anticancer effects are partly mediated via inducing tumor cells apoptosis by increasing the Bax/Bcl-2 ratio (Aas et al., 2015 ▶). Additionally, farnesiferolC isolated from the resin of *F. assa-foetida* L. exerted anti-angiogenic activity (Lee et al., 2010 ▶).

Mousavi et al. (2015) ▶ reported auraptene apoptotic effects in MCF-7 cell line (IC_50_ = 59.7 µM). They revealed that auraptene induced a sub-G1 peak in the flow cytometry histogram of treated cells compared to control cells. In this study, DNA fragmentation was suggested as one of the underlying mechanisms of auraptene-induced apoptosis. Also, western blot analysis showed that auraptene significantly up-regulated Bax expression in MCF-7 cells compared to untreated controls (Mousavi et al., 2015 ▶).

DAW22, a natural sesquiterpene coumarin isolated from *F. ferulaeoides* (Steud.) Korov. Induced C6 glioma cell apoptosis and endoplasmic reticulum (ER) stress, via mitochondrial and death-receptor-mediated pathways (Zhang et al., 2015 ▶).

Dietary phytochemicals present in *F. assa-foetida*, like luteoline, ferutinin and ferutidine, induced apoptosis and inhibited cell proliferation at the level of DNA synthesis (in S- phase) (Bansal et al., 2012 ▶; Matin et al., 2014 ▶). *F. assa-foetida* extract exerted anti-apoptotic activity in cerebellar granule neurons by induction of cell cycle arrest in G_0_/G_1_ phase; therefore, *F. assa-foetida* extract was suggested to be used against neurologic disorders (Tayeboon et al., 2013 ▶).

Gharaei et al. (2013 ▶) revealed that* F. gummosa *Boiss. extracts exerted anti-proliferative as well as apoptosis-inducing effects in a human gastric adenocarcinoma cell line (AGS). They also reported that *F. gummosa *extracts inhibited AGS cell line proliferation in a dose-dependent manner with IC_50 _values of 37.47 µg/mL for flower and 32.99 µg/mL for leaf extracts. *F. gummosa *extracts also induced apoptosis, as reflected by DNA fragmentation and plasma membrane translocation of phosphatidyl serine (Gharaei et al., 2013 ▶). *F. gummosa* flower and leaf extracts inhibited angiogenesis in a concentration-dependent manner (10-30 µg/ml), reflecting the possible presence of anti-angiogenic compounds (Mirzaaghaei et al., 2014 ▶).

In another study, it was reported that *F. assa-foetida* and *F. gummosa *exert cytotoxic effects. The cytotoxic effects of *F. assa-foetida *were mediated through three mechanisms including inhibition of mutagenesis, DNA destruction and cancer cell proliferation, while *F. gummosa *exerted its effects via cell cycle arrest and induction of apoptosis (Asadi-Samani et al., 2015 ▶).

Cytotoxic activity of sesquiterpene coumarins isolated from *F. nartex* was examined by Alam et al. (2016 ▶). These researchers reported that n-hexane fraction of *F. nartex* extract shows significant cytotoxic activity against PC3 cancer cells with an IC_50_ value of 5.43 ± 0.24 µg/ml (Alam et al., 2016 ▶). *F. vesceritensis *extract, as a new natural source of lapiferin, showed promising specific cytotoxic activity against human breast cancer cells. The cytotoxic activity was shown to be mediated through induction of apoptosis. Lapiferin evoked multiple pathways involving enhancement of DNA fragmentation, activation of caspases and induction of histone acetylation, all triggering apoptosis (Gamal-Eldeen and Hegazy, 2010 ▶). 

The ethyl acetate fraction of *F. sinkiangensis* extract revealed efficient inhibiting effects on tumor cells proliferation and enhanced the apoptosis rate in tumor cells (Zhang et al., 2015).

**Mechanisms of action**

It has been found that natural agents with cell-based properties can be divided into two categories of cytotoxic and/or anti-proliferative compounds (Keskin et al., 2000 ▶). For example, sesquiterpene coumarins isolated from the *Ferula* genus, showed both growth inhibitory and cytotoxic activities in different cancerous cell lines (Ryuet al., 2001 ▶).

Umbelliprenin has exerted anti-proliferative effects on M4Beu cells (human metastatic pigmented malignant melanoma cell line) through cell cycle arrest in G1 phase (Barthomeuf et al., 2008 ▶) and cytotoxic effects on A549 (human lung cancer cell line) via mitochondrial-dependent mechanisms (Barthomeuf et al., 2008 ▶; Khaghanzadeh et al., 2012 ▶).

It seems that two different mechanisms of cellular growth inhibition consist of lowering proliferation rate and induction of cellular death through apoptosis or necrosis.

Generally, Bcl-2 family proteins such as Bcl-2 protein and Bax protein, have important regulatory roles in apoptosis. Aldaghi et al. indicated that farnesiferol C and microlobin, two sesquiterpene coumarins isolated from *F. szowitsiana*, have greater binding affinity to Bax protein in comparison to Bcl-2 protein. These researchers assumed that the interaction between drugs and hydrophobic groove of Bax protein might result in conformational changes and insertion of Bax protein into mitochondrial membrane, consequently inducing Bax-dependent apoptosis (Aldaghi et al., 2016 ▶). In another study, RT-PCR analysis of *Bax* and *Bcl-2 *genes showed that dendrosomal form of farnesiferol C could suppress AGS cell proliferation, at least in part, via inducing apoptosis. Moreover, some recent research revealed that coumarin compounds could induce apoptosis by modulating Bax/Bcl-2 and caspase pathways (Gholami et al., 2013 ▶; Sadeghizadeh et al., 2008 ▶).

**Figure2:**
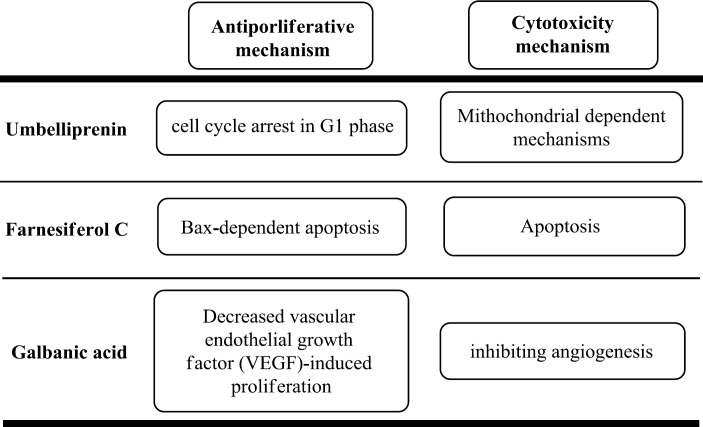
Overview of different mechanisms through which *Ferula*-isolated compounds decrease cellular growth

Cytotoxic activity of galbanic acid was mediated through inhibiting angiogenesis, the essential process required for tumor growth and metastasis. Galbanic acid significantly decreased vascular endothelial growth factor (VEGF)-induced proliferation and inhibited VEGF-induced migration and tube formation in human umbilical vein endothelial cells (HUVECs). These effects were accompanied by decreased phosphorylation of p38-mitogen-activated protein kinase (MAPK), c-Jun N-terminal kinase (JNK), and AKT, and decreased expression of VEGFR targets endothelial nitric oxide synthase (eNOS) and cyclin D1 in VEGF-treated HUVECs (Kim et al., 2011 ▶). In another study, galbanic acid showed a promising inhibitory activity against farnesyltransferase (FTase), an essential enzyme needed for tumor growth in pancreas and colon cancers (Figure 2) (Cha et al., 2011 ▶).

**Table 1:** Overview of the cytotoxic activities of *Ferula species*

**Plant Name**	**Important** **Compound**	**Biological activity**	**cell line**	**Tested concentrations** **(IC**_50_ **) µg/mL**	**Mechanism of action**	**Reference**
***F.vesceritensis***	Lapiferin	Cytotoxic Apoptotic	MCF7 MCF7	12.85 10	Anticancer activity Induction of apoptotic cell death through enhancement of DNA fragmentation, activation of caspases and induction of histone acetylation	Gamal-Eldeen and Hegazy, 2010
***F. assa-foetida***	8-acetoxy-5-hydroxy Umbelliprenin	Cytotoxic	A549	15.09	Potent and specific inhibition of NF-κB	Appendino et al., 2006
***F. assa-foetida***	Coumarin compounds	Cytotoxic	HepG2		Inhibition of mutagenesis, DNA destruction andcancer cells proliferation while increasing proteolyticenzymes activity	Asadisamani et al., 2015
***F. gummosa***	Sesquiterpenes, coumarins	Cytotoxic	HepG2		Induction of cell cycle arrest and apoptosis	Asadisamani et al., 2015
***F. assa-foetida***	Ferutinin	Cytotoxic	CT26 HT29	26 29	Induction of apoptosis	Arghiani et al., 2014
***F. communis***	Ferulenol	Cytotoxic	MCF-7	1	Reorganization of the microtubule network in MCF-7 cells and alteration of nuclear morphology	Altmann and Gertsch, 2006
***F. sinkiangensis***	Ethyl acetate Fraction	Cytotoxic	MCF7	9.0 mg/L	Inhibition of tumor cell proliferation	Zhang et al., 2015a
***F. lutea***	Methyl caffeate	Cytotoxic	HCT-116 IGROV-1 OVCAR-3	22.5±2.4 17.8±1.1 25±1.1	Not-mentioned	Znati et al., 2014a
***F.szowitziana***	Dendrosomal farnesiferol C	Antiproliferative and Apoptotic	AGS (gastric cancer)	>150 μΜ (24h) 80 μΜ (48h)	Significant time- and dose-dependent suppression of AGS cells proliferation	Aas et al., 2015
***F. assa-foetida***	kellerin	Antiviral	HSV-1	concentrations of 10, 5 and 2.5 μg/mL	Reduction of viral titre of the HSV-1 DNA viral strains KOS	Ghannadi et al., 2014
***F. pseudalliacea***	Kamolonol, 4′-hydroxy kamolonol acetate,and farnesiferon B	Cytotoxic	HeLa-60	3.8, 4.5, and 7.7μM, respectively	Seemingly, these compounds interfere with fundamental processes of growth and metabolism of the cells.	Dastan et al., 2014a
***F. lutea***	n-butanol extract	Cytotoxic	K562	40 μg/mL	Low cytotoxicity compared to doxorubicin.	Znati et al., 2014b
***F. szowitsiana***	Auraptene	Cytotoxic	MCF7	59.7 μM	Induction of a sub-G1 peak in the flow cytometry histogram, DNA fragmentation and apoptosis as well as up-regulation of Bax expression.	Mousavi et al., 2015
***F. szowitsiana***	Chimganin-Chimgin	Cytotoxic	MCF-7	45.2 for Chimginand 28 for Chimganin	Not-mentioned.	Sahranavard et al., 2009
***F. sinkiangensis***	DAW22	Apoptotic	C6 glioma cell	18.92 μM in 24h	Induction of apoptosis through ER stress and mitochondrial death-receptor mediated pathways.	Zhang et al., 2015b
***F. gummosa***	Ethanolic extract	Cytotoxic	BHY (human oral squamous	(0.001±1.2 mg/mL) in 72h	Induction of apoptosis and cell-cycle arrestin G1/S phase.	Gudarzi et al., 2014
***F. szowitsiana***	Umbelliprenin	Antigenotoxic	human lymphocytes	25 to 400 μM	Inhibition of H_2_O_2_-induced DNA damage.	Soltani et al., 2009
***F. ovina***	Ferutinin	Apoptotic	MCF7, TCC and HFF3	29, 24 and 36 μg/ml, respectively	Induction of apoptosis.	Matin et al., 2014
***F. szowitsiana***	Farnesiferol C	Antitumor	Human umbilical vein endothelial cells (HUVEC)	1 mg/kg body weight	Inhibition of VEGFR1.	Lee et al., 2010
***F. badrakema***	Mogoltacin	Increasing the Cytotoxicity of vincristine	TCC		Inhibition of P-glycoprotein-mediateddrug transport	BehnamRassouli et al., 2009
***F. pseudalliacea***	Sanandajin	Cytotoxic	HeLa cells	2.2 µM	Not mentioned.	Dastan et al., 2014b
***F. ovina***	Tschimgine	Acetylcholinesterase inhibitory effect	Red blood cell (RBC) AchE	(inhibition 63.5%)	Anti-cholinesterase activity	Karimi et al., 2010
***F. narthex***	Sesquiterpenecoumarins	Anticancer	PC3 cells	14.074±0.414μg/mL	Not mentioned.	Alam et al., 2016
***F. oopoda***	Dehydrooopodin	Cytotoxic	MCF7 and K562	15 and 5µM, respectively	Not mentioned.	Kasaian et al., 2014a
***F. assa-*** ***foetida***	Methanolic extract	Cytotoxic	MDA-MB-231 Cell Line	About 650 μg/mL In 72h	Not mentioned.	Vahabi et al., 2014
***F. gummsa***	Ethanolic extract	Cytotoxic	Gastric cancer, AGS	37.47 µg/mL	Induction of apoptosis via induction of DNA fragmentation and plasma membrane translocation of phosphatidyl serine.	Gharaei et al., 2013
***F. szowitsiana***	Umbelliprenin	Apoptotic	Jurkat T-CLL		Induction of caspase-mediated apoptosis. Activation of intrinsic and extrinsic pathways of apoptosis by activation of caspase-9 and caspase-8.	Gholami et al., 2013
***F. szowitsiana***	Umbelliprenin	Cytotoxic	QUDB and A549 lung cancer	47±5.3 μM and 52±1.97 μM, respectively	Induction of apoptosis.	Khaghanzadeh et al., 2012

## Conclusion

Ferula plants are rich sources of phytochemicals such as sesquiterpene coumarins, sesquiterpene lactones and sulfur-containing compounds. Over the last decade, considerable attention has been paid to investigate the potential cytotoxic activities of Ferula (Apiaceae) plants and their main constituents. This review aimed to highlight cytotoxic activities of Ferula species and their phytochemicals (Table 1). We also discussed different mechanisms through which active compounds isolated from Ferula species decrease cellular growth or induce cell death.

 It is assumed that the most prominent biological features of the genus Ferula are their cytotoxic effects. Previous reports proposed that Ferula phytochemicals have different activities. This probably suggests that much effort still remains to be made to identify potent and effective Ferula compounds that could be appropriate to be used as adjuvant therapy along with the conventional antibiotics. It is ultimately suggested that considering the versatile biological activities of sesquiterpene coumarins, these compounds may have an even broader range of biological applications in the future.
